# Expression characteristics of pineal miRNAs at ovine different reproductive stages and the identification of miRNAs targeting the *AANAT* gene

**DOI:** 10.1186/s12864-021-07536-y

**Published:** 2021-03-25

**Authors:** Ran Di, Qiu-Yue Liu, Shu-Hui Song, Dong-Mei Tian, Jian-Ning He, Ying Ge, Xiang-Yu Wang, Wen-Ping Hu, Joram-Mwashigadi Mwacharo, Zhang-Yuan Pan, Jian-Dong Wang, Qing Ma, Gui-Ling Cao, Hui-Hui Jin, Xiao-Jun Liang, Ming-Xing Chu

**Affiliations:** 1 Key Laboratory of Animal Genetics and Breeding and Reproduction of Ministry of Agriculture and Rural Affairs, Institute of Animal Science, Chinese Academy of Agricultural Sciences, No. 2, Yuanmingyuan West Rd, Beijing, 100193 China; 2 National Genomics Data Center & CAS Key Laboratory of Genome Sciences and Information, Beijing Institute of Genomics, Chinese Academy of Sciences, and China National Center for Bioinformation, Beijing, China; 3 Small Ruminant Genomics, International Center for Agricultural Research in the Dry Areas (ICARDA), Addis Ababa, Ethiopia; 4 Research Center of Grass and Livestock, NingXia Academy of Agricultural and Forestry Sciences, No. 590, East Yellow River Road, Yinchuan, 750002 China

**Keywords:** Sheep, Pineal gland, miRNAs, Anestrus, Breeding season

## Abstract

**Background:**

Many recent studies have shown that miRNAs play important roles in the regulation of animal reproduction, including seasonal reproduction. The pineal gland is a crucial hub in the regulation of seasonal reproduction. However, little is known about the expression characteristics of pineal miRNAs in different reproductive seasons (anestrus and breeding season). Therefore, the expression profiles and regulatory roles of ovine pineal miRNAs were investigated during different reproductive stages using Solexa sequencing technology and dual luciferase reporter assays.

**Results:**

A total of 427 miRNAs were identified in the sheep pineal gland. Significant differences in miRNA expression were demonstrated between anestrus and the breeding season in terms of the frequency distributions of miRNA lengths, number of expressed miRNAs, and specifically and highly expressed miRNAs in each reproductive stage. KEGG analysis of the differentially expressed (DE) miRNAs between anestrus and the breeding season indicated that they are significantly enriched in pathways related to protein synthesis, secretion and uptake. Furthermore, transcriptome analysis revealed that many target genes of DE miRNAs in the ribosome pathway showed relatively low expression in the breeding season. On the other hand, analyses combining miRNA-gene expression data with target relationship validation in vitro implied that miR-89 may participate in the negative regulation of aralkylamine N-acetyltransferase (*AANAT*) mRNA expression by targeting its 3’UTR at a unique binding site.

**Conclusions:**

Our results provide new insights into the expression characteristics of sheep pineal miRNAs at different reproductive stages and into the negative regulatory effects of pineal miRNAs on *AANAT* mRNA expression.

**Supplementary Information:**

The online version contains supplementary material available at 10.1186/s12864-021-07536-y.

## Background

MicroRNAs (miRNAs) belong to a large family of endogenous noncoding RNAs (ncRNAs). MiRNAs may regulate the expression of target genes by binding to complementary regions in their 3′ untranslated regions (3’UTRs) [1, 2]. Many studies have shown that miRNAs play important regulatory roles in animal reproduction [3–9]. In recent years, miRNAs have also been found to be involved in the regulation of animal seasonal reproduction [10–13]. The pineal gland is a key organ known to transduce the photoperiod signal to the reproductive axis and is therefore crucial for seasonal reproduction [14, 15]. However, little is known about the expression characteristics of pineal miRNAs in different reproductive seasons (nonbreeding and breeding seasons). Sheep are regarded as a typical animal model for seasonal reproduction studies [16–20]. Therefore, one objective of this study was to reveal the expression characteristics of pineal miRNAs in sheep during different reproductive stages. The second purpose of this study was to screen differentially expressed miRNAs among the different reproductive stages and predict their probable functions in the pineal gland.

The joint analysis of miRNAs and gene expression profiles has become feasible in recent years [21–24], and miRNA-target gene pairs can be predicted. Furthermore, the specific target sites at which miRNAs regulate gene expression can also be revealed by a double luciferase reporter assay [25, 26]. The pineal gland participates in the regulation of seasonal reproduction mainly through melatonin [27–30]. Known rate-limiting enzymes for melatonin synthesis include aralkylamine N-acetyltransferase (AANAT) and hydroxyindole O-methyltransferase (HIOMT). Thus, the third objective of this study was to identify the miRNAs targeting the genes encoding the above-mentioned rate-limiting enzyme that were differentially expressed during the different reproductive stages. Together, this study provides new insights into the expression characteristics and roles of pineal miRNAs in sheep during different reproductive stages.

## Results

### Expression characteristics of pineal small RNAs in sheep at different reproductive stages

**Fig. 1:**
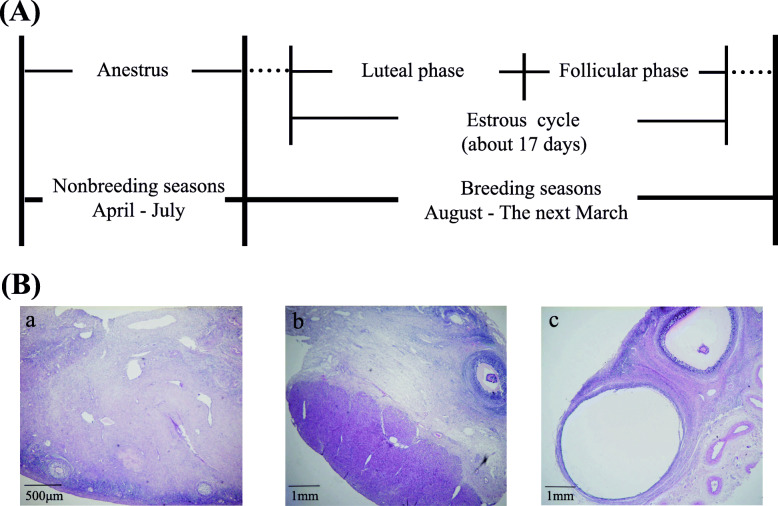
Seasonal reproductive characteristics of Tan sheep (A) and ovarian sections of Tan sheep at different sampling stages in this study (B). (A) The anestrus season is usually from April to July for Tan sheep, and the other months are the breeding season. In the breeding season, every estrous cycle is approximately 17 days, including the luteal phase and follicular phase. (B) In ovarian sections from anestrous ewes, no large corpus luteum or follicles were observed (a). An obvious corpus luteum with a diameter of more than 5 mm was observed on the ovary surface at the luteal phase (b), and a large antral follicle was found in the follicular phase (c)

**Fig. 2:**
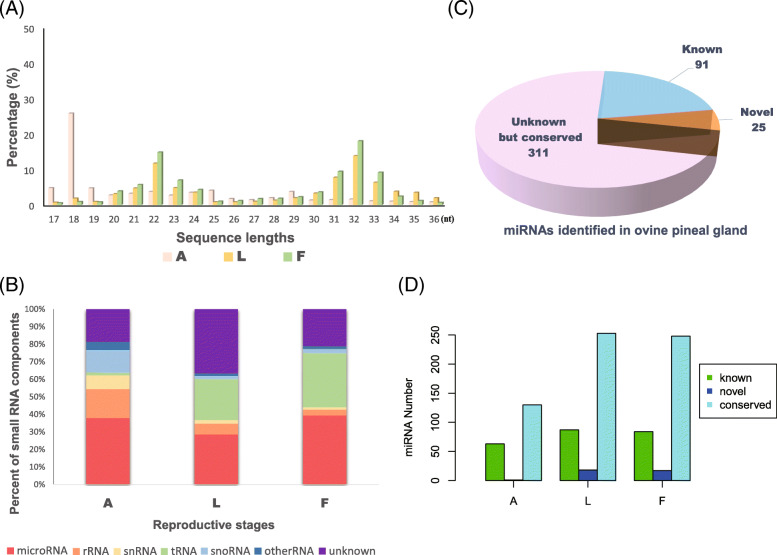
Expression characteristics of pineal small RNAs in sheep at different reproductive stages. **a** Distribution of sequence lengths at different reproductive stages based on the abundance of clean reads. X axis: sequence lengths; Y axis: Percentage of reads number with each length. A: anestrus; L: luteal phase; F: follicular phase. **b** The composition of RNA classes at different reproductive stages. **c** The number of expressed miRNAs in the ovine pineal gland, including known, conserved and predicted novel miRNAs. d The number of expressed miRNAs at different reproductive stages

Pineal tissues were collected at different reproductive stages, and the accuracy of the sampling stage determination was confirmed by the results obtained from ovarian sections (Fig. 1). To reveal the expression patterns of pineal small RNAs in different ovine reproductive stages, high-throughput deep sequencing was used to obtain the pineal sRNA expression profiles at each stage. Generally, the clean reads in the pineal gland showed a three-peaked length distribution, with peaks at 18, 22 and 32 nucleotides (nt) (Fig. 2a). The majority of expressed reads from the anestrus sample were 18 nt long, whereas reads from samples during the breeding season were predominantly 22 or 32 nt long (Additional file 1). To further assess the efficiency of high-throughput sequencing in detecting miRNA, all the mapped clean reads (clean read counts and mapping ratios are listed in Additional file 2) were annotated and classified by alignment against ncRNAs. Among the diverse sequences of ovine pineal ncRNAs (including miRNAs, rRNAs, sRNAs, tRNAs and other RfamRNAs), the proportion of miRNAs was always the highest in each stage (Fig. 2b), and their values were also similar among different stages. However, the proportions of rRNAs, sRNA and other RfamRNA were relatively higher during anestrus than during the breeding season. In total, 427 miRNAs were identified in the ovine pineal gland, including 91 known miRNAs, 311 conserved miRNAs and 25 predicted novel miRNAs (Fig. 2c). Compared with the two stages (luteal and follicular phases) in the breeding season, expressed miRNAs (including known, conserved and novel miRNAs) were the least abundant in ovine anestrus (Fig. 2d).

Next, the functions of miRNAs that were specifically expressed in anestrus or the breeding season were predicted. KEGG pathway analysis (Additional file 3) showed that the target genes of miRNAs that are expressed specifically in anestrus were predominantly enriched in endocytosis, mTOR and MAPK signaling pathways. These pathways are mainly associated with hormone uptake, protein synthesis, and cell proliferation and differentiation. On the other hand, the target genes of miRNAs that were expressed specifically in the breeding season were predominantly involved in pathways such as the mTOR signaling pathway, apoptosis and axon guidance (Additional file 3). These pathways are mainly associated with protein synthesis, cell growth and death, and the formation of neuronal networks.

Meanwhile, the expression of miRNAs was also ranked in each reproductive stage, and the 20 most highly expressed miRNAs are displayed in Table 1. The results indicated that the top 20 miRNAs were similar between the two stages (luteal and follicular phases) in the breeding season; however, they were significantly different between the breeding season and anestrus. In anestrus, miR-142 (homology ID: aca-miR-5441) was the most abundant miRNA, accounting for 86% of the total expressed miRNA. KEGG analysis showed that the target genes of miR-142 were predominantly enriched in oxidative phosphorylation, glycerolipid metabolism and phosphatidylinositol signaling pathways. In addition to miR-142, high expression of miR-202 (homology ID: tae-miR-5086) and miR-2 (homology ID: cel-miR-51-5p) was also observed during anestrus. Oar-miR-181a, Oar-miR-26a and Oar-miR-143 showed the highest levels of expression in the breeding season. Additionally, Oar-let-7a was highly expressed in all reproductive stages.

**Table 1:** Top 20 miRNAs expressed in the sheep pineal gland at each reproductive stage

Rank	Anestrus	Luteal phase	Follicular phase
1	miR-142	oar-miR-181a	oar-miR-181a
2	miR-202	oar-let-7a	oar-miR-26a
3	oar-let-7a	oar-miR-26a	oar-let-7a
4	miR-2	oar-miR-143	oar-miR-143
5	oar-miR-181a	miR-1	oar-miR-30d
6	miR-3	oar-let-7f	oar-miR-30a-5p
7	miR-18	miR-154	miR-154
8	miR-21	miR-175	miR-175
9	miR-1	oar-miR-30d	oar-miR-22-3p
10	oar-let-7f	oar-let-7 g	miR-6
11	oar-let-7 g	oar-let-7c	miR-7
12	oar-miR-143	oar-miR-21	miR-1
13	oar-miR-26a	oar-miR-30a-5p	oar-let-7f
14	oar-miR-99a	miR-6	oar-let-7c
15	oar-miR-191	miR-7	oar-let-7 g
16	oar-miR-21	miR-5	miR-4
17	oar-let-7c	miR-2	miR-5
18	miR-200	oar-miR-22-3p	miR-2
19	miR-7	oar-let-7i	oar-let-7i
20	oar-miR-30a-5p	miR-4	miR-14

### Differentially expressed (DE) miRNAs among different ovine reproductive stages and their probable functions in the pineal gland

**Fig. 3:**
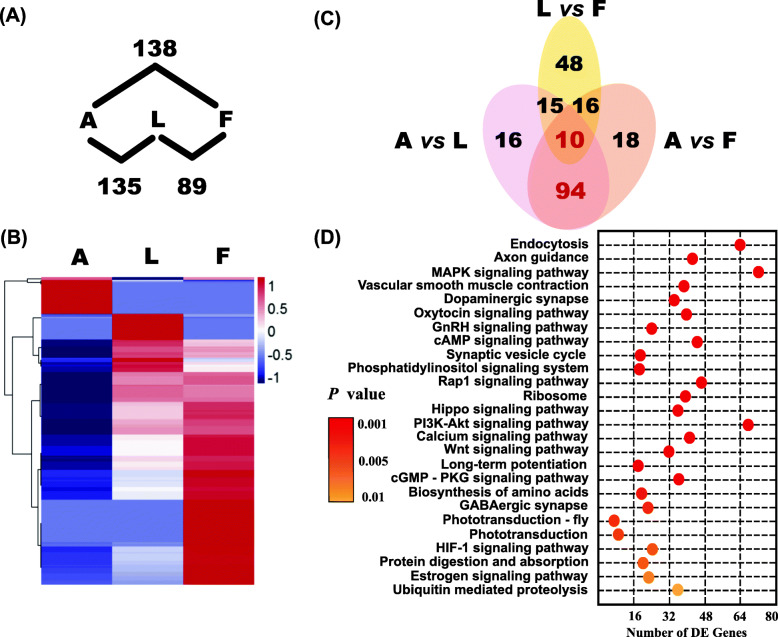
Outline of differentially expressed miRNAs among different reproductive stages.**a** Number of differentially expressed (DE) miRNAs detected among three stages. DE miRNAs were identified with the edgeR software package (version: 3.12). **b** Dendrogram of hierarchical clustering of expressed miRNAs among three reproductive stages. The clustering analysis was performed by pheatmap (v1.0.2). **c** Venn diagram showing the overlap of differentially expressed miRNAs among three comparisons (A vs. L; L vs. F; A vs. F). **d** Pathways in which the target genes of differentially expressed miRNAs between anestrus and the breeding season were mainly enriched. The color of the circle represents the *P* value at which a certain pathway is enriched. X axis: number of differentially expressed genes in the specific KEGG pathway. The KEGG pathways were analyzed by clusterProfiler package (v3.16.0)

We determined the DE miRNAs among three reproductive stages (anestrus, follicular phase and luteal phase). The largest number of DE miRNAs was detected between anestrus and the follicular phase (Fig. 3a). Hierarchical clustering of miRNAs (Fig. 3b) also indicated that the differences in miRNA expression between anestrus and the follicular phase were greatest among the three stages analyzed. Furthermore, the majority of the DE miRNAs between anestrus and the two stages of the breeding season overlapped (Fig. 3c). Therefore, these overlapping miRNAs could be considered DE miRNAs between anestrus and the breeding season. To determine the probable biological functions of the overlapping miRNAs, we performed a pathway analysis of the target genes of these miRNAs. The analysis revealed that these miRNAs were mainly enriched in pathways related to protein biosynthesis, secretion and uptake (such as biosynthesis of amino acids, ribosome, cAMP signaling pathway, vascular smooth muscle contraction, axon guidance, dopaminergic synapses, and endocytosis pathway) and the phototransduction pathway (*P* < 0.01) (Fig. 3d, Additional file 4). Moreover, the results of the transcriptome analysis (Additional file 5) showed that the majority of the target genes in these pathways exhibited differential expression between the seasons. For example, *RPLP1*, *RPLP2*, *RPL18A*, *RPL35*, *RPS5*, *RPS13* and *RPSA* in the ribosome pathway showed significantly lower expression levels in the breeding season than in anestrus (Additional file 6).

In addition, the overlapping differentially expressed genes between anestrus and the luteal phase and between anestrus and the follicular phase were also screened out to represent the expression differences in genes between nonbreeding and breeding seasons. The highly expressed genes during anestrus and the breeding season in the pineal gland of sheep are shown in Additional file 7. Some of the highly expressed genes in anestrus were related to protein synthesis and hormone secretion. Highly expressed genes in the breeding season were involved in the ribosome pathway, cAMP signaling pathway and other pathways.

### Prediction of important miRNA–target gene pairs

**Fig. 4:**
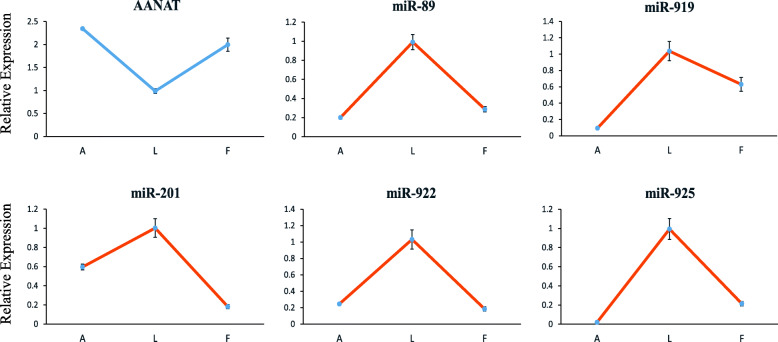
Results of real-time quantitative PCR for the *AANAT* gene and five miRNAs. Data are the mean ± SEM from three ewes in each group

The joint analysis of negatively correlated miRNA–mRNA pairs and miRNA target binding prediction has been demonstrated to be a feasible approach for predicting miRNA-target gene pairs [31, 32]. We therefore measured pineal mRNA profiles at different reproductive stages to examine miRNA–mRNA correlations at the whole-genome scale. Among the negatively correlated pairs, many miRNA-target gene pairs with binding sites were predicted. We first investigated the transcriptional regulatory role of miRNAs on key rate-limiting enzyme genes in melatonin synthesis. The expression of *AANAT* mRNA showed significant variation at different reproductive stages (Fig. 4). Therefore, the miRNAs that were significantly and negatively correlated with the *AANAT* expression pattern were predicted and summarized in Table 2. To validate the results from high-throughput sequencing, real-time quantitative PCR was performed for the five miRNAs in Table 2 and the *AANAT* gene. The results of quantitative PCR (Fig. 4) were consistent with the sequencing data, and these miRNAs exhibited an inverted expression to that of the *AANAT* gene. Additionally, for the expression of *HIOMT* mRNA, there was no significant difference among the reproductive stages. Therefore, miRNAs targeting the gene were not predicted in this study. In addition to *AANAT*, miRNAs potentially targeting several differentially expressed genes in the ribosome pathway were also predicted (Additional file 8).

**Table 2:** List of miRNAs that are negatively correlated with *AANAT* expression in the sheep pineal gland

miRNA ID	Conserved ID	Mature sequence	Correlation coefficient R	Predicted target relationships
miR-89	hsa-miR-214-3p	acagcaggcacagacaggcag	−0.424	Yes
miR-69	mmu-miR-326-3p	ucucugggccugugucuuaggcu	−0.420	Yes
oar-miR-25	hsa-miR-25-3p	cauugcacuugucucggucuga	−0.378	Yes
hsa-miR-28-5p	hsa-miR-28-5p	aaggagcucacagucuauuga	−0.297	Yes
oar-miR-370-5p	hsa-miR-370-3p	gccugcugggguggaaccuggu	−0.289	Yes
miR-58	mmu-miR-744-5p	ugcggggcuagggcuaacagca	−0.289	Yes
miR-201	hsa-miR-3156-3p	uucccacucccucuguccgccu	−0.960	No
miR-919	–	gaggguuuggguuuggucguggga	−0.955	No
miR-922	–	uccccccacgcccgggcca	−0.955	No
miR-925	–	gggcaggguugggagggu	−0.955	No
miR-918	bta-miR-2285b	aaaaucugaacaaacuuucugg	−0.849	No
oar-miR-136-5p	mmu-miR-136-5p	acuccauuuguuuugaugaug	−0.830	No
oar-miR-218a	hsa-miR-218-5p	uugugcuugaucuaaccaug	−0.786	No
oar-miR-411b-5p	mmu-miR-412-5p	uggucgaccauaaaacguacgu	−0.763	No
miR-325	–	uacugugccacggauggguagc	−0.742	No
miR-355	–	uacugugccacggauggguagc	−0.742	No
miR-78	mmu-miR-542-3p	ugugacagauugauaacugaaa	−0.690	No
miR-643	mmu-miR-873a-5p	gcaggaacuugugagucuccu	−0.684	No
miR-88	ssc-miR-7134-5p	auguccgcggguucccuauccc	−0.680	No
miR-81	hsa-miR-424-3p	caaaacgugaggcgcugcuaua	−0.674	No
miR-30	mmu-miR-146a-5p	ugagaacugaauuccauaggcugu	−0.657	No
miR-52	hsa-miR-4456	ucuggugggaaggaagggac	−0.655	No
oar-miR-10a	cel-miR-57-5p	uacccuguagauccgaauuugu	−0.650	No
miR-166	mmu-miR-184-3p	uggacggagaacugauaagg	−0.648	No
miR-205	hsa-miR-3199	agggacugaggcgugagccu	−0.623	No
miR-245	–	cccgguacugagcugacccgagc	−0.612	No

### Validation of the target relationships between *AANAT* and candidate miRNAs

**Fig. 5:**
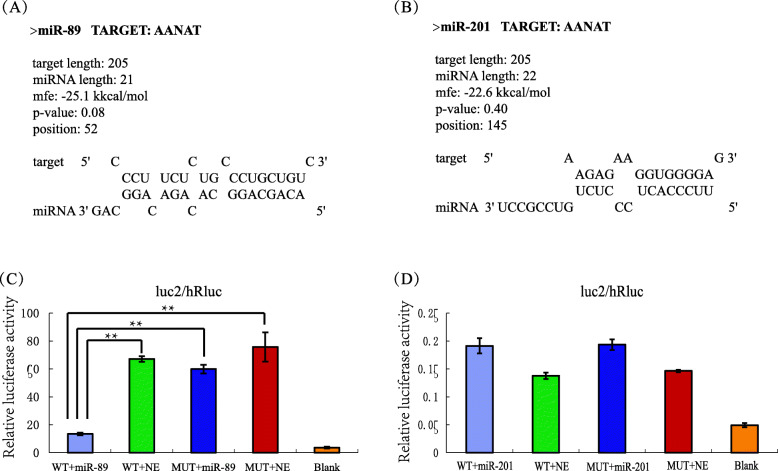
Results of prediction and validation of the target relationships between miRNAs and the 3’UTR of the *AANAT* gene. The target sites of miR-89 (**a**) and miR-201 (**b**) in the 3’UTR of the *AANAT* gene were predicted by RNAhybrid software. The results of validation of the target relationships of miR-89 and miR-201 through the dual luciferase reporter system are shown in (**c**) and (D). WT + miR-89: cells cotransfected with pmirGLO-AANAT wild-type 3’UTR (0.025 μg) and pcDNA6.2-GW/miRNA-89 (0.075 μg); WT + NE: cells cotransfected with pmirGLO-AANAT wild-type 3’UTR (0.025 μg) and pcDNA6.2-GW empty vector (0.075 μg); MUT + miR-89: cells cotransfected with pmirGLO-AANAT mutant-type 3’UTR (0.025 μg) and pcDNA6.2-GW/miRNA-89 (0.075 μg); MUT + NE: cells cotransfected with pmirGLO-AANAT mutant-type 3’UTR (0.025 μg) and pcDNA6.2-GW empty vector (0.075 μg); blank: cells without transfected vector. The test groups for miR-201 were similar to those for miR-89. **: *P* < 0.01

**Fig. 6:**
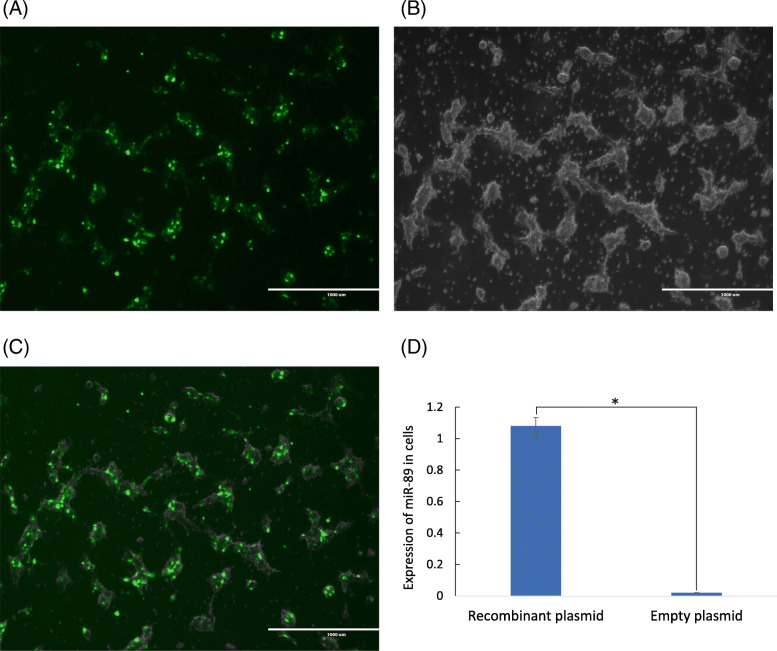
Efficiency of miRNA transfection in cells. Expression of green fluorescent protein gene in the pcDNA6.2-GW/miRNA recombinant plasmid was detected after transfection for 24 h (magnification: 4×, scale bars = 1000 μm; **a**: fluorescent detection image, **b**: bright field image, **c**: merged fluorescent image). **d**. Compared with the empty plasmid group, the expression levels of miR-89 were significantly higher in cells with the pcDNA6.2-GW/miR-89 recombinant plasmid

To further verify the target relationships between *AANAT* and candidate miRNAs, one miRNA (miR-89) that was predicted to show a target relationship (completely binding) and one (miR-201) that was predicted to show just partial binding but exhibited a high negative correlation coefficient were selected for validation in vitro. Among the miRNAs with a complete binding relationship, miR-89 was selected for validation because it was previously detected in sheep (defined as Oar-miR-214-3p) and suggested to be involved in the regulation of breeding activities [33]. Next, the 3′ UTR sequence of the *AANAT* gene in Tan sheep was obtained, and the sequence was consistent with the corresponding region of the reported *AANAT* mRNA sequence (NM_001009461.1) in a cross of the Dorset × Rambouillet sheep [34, 35]. The predicted target site of miR-89 in the 3’UTR of the *AANAT* gene is shown in Fig. 5a. The possible binding site of miR-201 with the 3’UTR of *AANAT* was also predicted by RNAhybrid software. The 2nd and 8th bases at the 5′ end of the miRNA showed semibinding states with the 3’UTR of *AANAT* (Fig. 5b). Then, the dual luciferase reporter system was used to further verify the targeting role of the miRNAs associated with the 3’UTR of *AANAT* in vitro. The miRNAs were successfully transfected into 293FT cells, and the efficiency of transfection is indicated in Fig. 6. As shown in Fig. 5c, the relative luciferase activity of luc2/hRluc in the group cotransfected with miR-89 and the wild-type 3′ UTR of *AANAT* was significantly lower (*P* < 0.01) than in the group transfected with the wild-type 3′ UTR of *AANAT* alone or the group cotransfected with miR-89 and the mutant-type 3′ UTR of *AANAT*. This result verified that miR-89 can target the 3′ UTR of *AANAT* and that the binding site is unique. Taken together, the results of validation of the target relationship and the observed expression of miRNA and *AANAT* mRNA during different stages implied that miR-89 participates in the negative regulation of *AANAT* mRNA expression by targeting its 3′ UTR. However, the data demonstrated that miR-201 had no apparent target effect on the 3′ UTR of *AANAT* (Fig. 5d). Therefore, the results of in vitro analysis demonstrated that the predicted miRNA–target mRNA pairs were accurate.

## Discussion

Many recent studies have shown that miRNAs play important roles in the regulation of reproduction [3–9], including seasonal reproduction [10–13]. The pineal gland is a crucial organ for seasonal reproduction. However, little is known about the expression characteristics of pineal miRNAs in different reproductive seasons. In this study, we investigated the miRNA expression profiles of the ovine pineal gland for the first time.

First, a higher number of expressed miRNAs were detected in the pineal gland of sheep compared with those identified miRNAs in zebrafish [22] and rats [36, 37]. Second, the results suggested that there are some differences in the expression characteristics of miRNAs between reproductive stages. Our previous and current results have demonstrated that the number of expressed miRNAs is lowest during anestrus in both the ovary [10] and pineal gland of sheep. Additionally, the most highly expressed miRNAs were also distinct among reproductive stages. Specifically, miR-142 was detected as the most highly expressed miRNA during anestrus. Similarly, high expression of miR-142 was also observed in the gonad during ovine anestrus [10]. Previous studies revealed the roles of miR-142 in suppressing proliferation, inducing apoptosis [38, 39] and regulating neurotransmission [40]. In this study, the synaptic vesicle cycle was one of the enrichment pathways for miR-142 target genes, which is related to neurotransmission. In addition, the enriched pathways (oxidative phosphorylation, glycerolipid metabolism and phosphatidylinositol signaling system) for the target genes of miR-142 were mainly associated with energy and lipid metabolism. Previous studies observed that the volume and number of mitochondria and lipid droplets in pinealocytes of seasonal breeders varied between short and long photoperiods [41, 42], implying the existence of differences in energy and lipid metabolism in pinealocytes between different seasons for seasonal breeders. In addition to miR-142, miR-202 is the second ranked miRNA that is expressed in anestrus. Several studies have shown, such as miR-142, that miR-202 is involved in cell proliferation [43–45]. For miR-26, which was highly expressed in the pineal gland of the breeding season, previous studies have suggested that it may play a role in the transition of the reproductive stages in goats [46], and its high expression was also detected in brain tissues of beef cattle [47]. For Oar-let-7a, which was highly expressed in each reproductive stage, several studies have shown that it is crucial for reproduction in the ovaries of sheep during different reproductive stages [10, 48]. Specifically, let-7 is involved in regulating important aspects of reproduction, including steroidogenesis [49] and follicular development [50]. In addition, miR-7 was listed in the top 20 expressed genes of the three stages simultaneously. miR-7 has been shown to regulate melatonin synthesis by acting as a linking molecule between leptin and norepinephrine signaling pathways in pig pineal [51].

A clear difference in miRNA expression existed between anestrus and the breeding season, as revealed in Fig. 3, implying distinct roles for pineal miRNAs between the seasons. KEGG analysis indicated that the target genes of both miRNAs that were expressed specifically during anestrus or the breeding season and the DE miRNAs between anestrus and the breeding season were highly enriched in pathways that were related to protein synthesis, secretion and uptake. Furthermore, based on the results of transcriptome analysis, many target genes of DE miRNAs in the ribosome pathway showed relatively low expression in the breeding season, which might lead to a change in protein synthesis. Previous studies indicated that the morphology of Golgi bodies and endoplasmic reticulum, which represent the ultrastructural characteristics of protein synthesis, showed seasonal-specific changes in the pinealocytes of seasonal breeders. For instance, when exposed to an artificially long photoperiod, rams exhibited some morphological changes in the endoplasmic reticulum and microtubules, and the volumes of mitochondria and lipid droplets in pinealocytes increased compared to rams under naturally short photoperiods [41]. Our results also suggest that protein synthesis in pinealocytes is more active under long photoperiods. However, for long-day breeders, the cisternae of the endoplasmic reticulum and Golgi bodies tend to dilate under short photoperiods in ground squirrels [52], and the Golgi body-endoplasmic reticulum-lysosome complex will be clearer under short photoperiods in Parakeet [42]. These studies, together with ours, suggest that protein biosynthesis in pinealocytes has seasonal features in seasonal breeders, and miRNAs might participate in the regulation of this variation.

Additionally, our study demonstrated that *AANAT* mRNA expression varied and may be regulated by miRNAs during different stages of reproduction. To date, few reports have addressed the differences in *AANAT* gene expression in the pineal glands of seasonal breeders among different reproductive stages. In the estrus cycle, it was found that the expression levels of *AANAT* in oocytes were higher in the follicular phase than in the luteal phase in rat ovaries [53], which is similar to the trend of *AANAT* variation in the pineal gland observed in this study. Analyses that have combined miRNA-gene sequencing data with target relationship validation in vitro have become popular in recent years [54, 55]. Using such analysis, it was found that miR-483 could negatively regulate *AANAT* expression in the rat pineal gland during development [37] and that the upregulation of miR-325-3p suppressed pineal *AANAT* expression after neonatal hypoxia–ischemia brain injury in rats [56]. In this study, the joint analysis of the expression of *AANAT* and miRNA, along with validation testing in vitro, suggested that miR-89 may negatively regulate *AANAT* mRNA expression at different reproductive stages by targeting its 3’UTR. Meanwhile, the miRNA without a predicted complete target relationship with the *AANAT* gene was proven to be unable to target the 3’UTR of the gene.

## Conclusion

The expression characteristics of pineal miRNAs in different stages of reproduction were revealed for the first time using domestic sheep. Significant differences in miRNA expression were demonstrated between anestrus and the breeding season. KEGG analysis of differentially expressed miRNAs between anestrus and the breeding season indicated that these miRNAs are significantly enriched in pathways related to protein synthesis, secretion and uptake. Furthermore, the transcriptome analysis revealed that many target genes of DE miRNAs in the ribosome pathway were expressed at relatively low levels in the breeding season. On the other hand, analyses combining miRNA-gene expression data with target relationship validation in vitro implied that miR-89 may participate in the negative regulation of *AANAT* mRNA expression by targeting its 3’UTR at a unique binding site.

## Methods

### Sample collection and deep sequencing

The Tan sheep in northern China are a typical seasonal breeder with a long anestrus period, which usually extends from April to July [10]. Therefore, the Tan sheep used in this study were selected from the Yanchi Tan sheep conservation farm in the Ningxia Autonomous Region, China. First, nine healthy and nonpregnant Tan ewes (three years old, body weight: 45–50 kg) were examined daily for estrous activity with a teaser ram during anestrus and the breeding season of 2016. Anestrus was judged to be a period without obvious signs of estrus for more than 36 days (equivalent to the period of two estrous cycles). On the 50th day during anestrus, three ewes (anestrus, *n* = 3) were simultaneously euthanized in a separate room. Before euthanasia, the overdose of pentobarbital (a barbiturate which depresses the central nervous system causing respiratory and cardiac arrest) and phenytoin (an antiepileptic drug which further decreases the central nervous system via sodium and calcium channel blockade) were administered at 0.22 mL/kg (85.8 mg/kg of pentobarbital and 11 mg/kg of phenytoin) over 10 s referring to the previous report [57]. Within 15 min after death, the entire pineal gland of ewes was removed from the brain and immediately collected in liquid nitrogen. Meanwhile, their ovaries were placed in 4% paraformaldehyde fixative to prepare paraffin sections in order to verify the accuracy of the sampling stages. Ovarian paraffin sections were stained with hematoxylin-eosin. In the breeding season, an estrous cycle includes the luteal and follicular phases. The date of estrus was recorded as the 1st day in each estrous cycle. Two continuous estrous cycles were recorded. The mean estrus cycle among six Tan ewes was 17.33 ± 0.26 days. Therefore, in the third estrous cycle, ewes were euthanized using the same method as above on the 9th day (luteal phase, *n* = 3) and during estrus (follicular phase, *n* = 3), and their pineal glands and ovaries were collected immediately as described above. Total RNA extraction and the construction of small RNA and mRNA libraries were performed using the same method as that in the literature [10]. The Illumina HiSeq 2000 system was used to sequence the library products. The sequence datasets have been deposited in the BioProject (Biological Project Library) database of Genome Sequence Archive [58] in National Genomics Data Center [59], Beijing Institute of Genomics (China National Center for Bioinformation), Chinese Academy of Sciences, with the accession number PRJCA000331.

### Bioinformatic analysis for small RNAs and mRNA

For miRNA analysis, clean reads of 17 ~ 36 bp were obtained using a method we described in a previous study [10]. Then, all clean reads were collapsed into tags based on sequence identity using an in-house built pipeline [60]. The unique tags were aligned to the Ovine reference genome (v3.1) by the mapper.pl of miRDeep2 (version 2.0.0.8) (no nucleotide changes; genome hits were less than 5), and then the miRNAs were predicted with the mirDeep2.pl program [61]. By comparison with the known ncRNAs (rRNAs, tRNAs, snRNAs, and snoRNA) from the NCBI GenBank and Rfam10.1 database, all the mapped tags were classed and annotated. In addition, using miRBase v21 [62, 63], all predicted miRNAs were classified as novel, known and conserved miRNAs using a method we previously described [10]. Finally, the expression levels of miRNAs and their difference between different reproductive stages were analyzed with the same method as in our previous study [10]. For the mRNA, quality control of the raw data, mapping of high-quality reads and expression analysis of genes were performed using a method we previously described [10]. Genes with an FDR < 0.05 were defined as differentially expressed genes [64].

Based on the 3′-UTR sequences that were extracted according to gene annotation information, miRNAs potentially targeting candidate genes were simultaneously predicted using the miRanda and RNAhybrid algorithms. Pairs of DE miRNAs and genes showing negative correlations were identified using R software. The function of target genes was predicted by KEGG pathway analysis. The enrichment *p*-values were calculated using a hypergeometric distribution.

### Validation of the expression of *AANAT* and its associated miRNAs

Real-time quantitative PCR (Q-PCR) was used to quantify the expression levels of *AANAT* mRNA and miRNAs that were randomly selected among those that were negatively correlated with *AANAT* in sequencing data. The stem-loop primers employed in RT-PCR of miRNAs and the primers employed in Q-PCR of *AANAT* and miRNAs are summarized in Additional file 9. Reverse transcription and Q-PCR were performed with the same method as in our previous study [10]. The data were analyzed with the 2^–ΔΔCt^ method [65].

### Validation of the target relationship between candidate miRNAs and *AANAT*

The 3′ UTR sequence of the *AANAT* gene of Tan sheep was obtained using the 3′-Full RACE Core Set with PrimeScript™ RTase kit (TaKaRa) and the specific primers GSP1 (5′-AGCGTCCACTGCCTGAAACC-3′) and GSP2 (5′-AGGTTTGGCTTCCATCCCG-3′). Following sequencing, the potential target site bound by candidate miRNAs was predicted using miRanda and RNAhybrid algorithms. For candidate miRNA-gene pairs, both a miRNA expression vector (pcDNA6.2-GW/EmGFP-miR) and a reporter vector (pmirGLO) in which the *AANAT* 3’UTR was fused to a firefly luciferase reporter were obtained to perform the dual luciferase reporter assay. The sequence that was inserted in the pcDNA6.2-GW/EmGFP-miR vector was designed based on the mature sequence of the miRNA (Additional file 10) and was synthesized by Invitrogen Trading (Shanghai) Company. The *AANAT* 3’UTR sequence that was inserted into the pmirGLO vector was synthesized by Shanghai Generay Biotech Company. The two vectors were cotransfected into 293FT cells using Lipofectamine 3000. The luciferase activity of luc2/hRluc in transfected cells will be significantly reduced when the miRNA targets the 3’UTR of the candidate gene. To determine whether there were other target sites besides the predicted one, a mutant-type 3’UTR of *AANAT* mutated at the predicted target site was also designed.

## Supplementary Information

**Additional file 1.** Percentage of reads number with different lengths at three reproductive stages of sheep.

**Additional file 2.** Expression information of small RNA reads in ovine pineal gland.

**Additional file 3.** The signaling pathways which the stage-specific expressed miRNAs were predominantly enriched in.

**Additional file 4 **The genes number and *P* value of pathways which target genes for differentially expressed miRNAs between anestrus and breeding season were enriched in.

**Additional file 5.** The expression level of target genes for DE miRNAs between anestrus and breeding season in different reproductive stages.

**Additional file 6.** Expression pattern of several genes in the ribosome pathway during different reproductive stages. (EPS 16678 kb)

**Additional file 7.** Highly expressed genes during anestrus or breeding season in pineal gland of sheep.

**Additional file 8.** Predicted miRNAs potentially targeting genes in ribosome pathway.

**Additional file 9 **Stem-loop primers employed for RT-PCR of miRNAs and primers employed for Q-PCR of *AANAT* and miRNAs.

**Additional file 10.** Mature sequences of miRNAs and inserted sequences in the pcDNA6.2-GW/EmGFP-miR vector.

## Data Availability

The raw datasets generated during the current study are available in the BioProject (Biological Project Library) database at National Genomics Data Center, Beijing Institute of Genomics (China National Center for Bioinformation) with the accession number PRJCA000331.

## Abbreviations

DE: Differentially expressed;
UTR: Untranslated region;
AANAT: Aralkylamine N-acetyltransferase
HIOMT: Hydroxyindole O-methyltransferase
ncRNAs: non-coding RNAs
