# Supplementary material for: Expression characteristics of pineal miRNAs at ovine different reproductive stages and the identification of miRNAs targeting the AANAT gene
Source: BMC Genomics. 2021 Mar 25;22:217. doi: 10.1186/s12864-021-07536-y (PMC7992348; doi:10.1186/s12864-021-07536-y)
Supplement: Supplementary file 1 — Additional file 1. Percentage of reads number with different lengths at three reproductive stages of sheep. [file 12864_2021_7536_MOESM1_ESM.docx]

**Additional file 1.** Percentage of reads number with different lengths at three reproductive stages of sheep.

| Sequence lengths (nt) | Percentage (%) | | |
| --- | --- | --- | --- |
|  | Anestrus | Luteal phase | Follicular phase |
| 17 | 4.79 | 0.71 | 0.53 |
| 18 | 25.84 | 1.84 | 0.9 |
| 19 | 4.74 | 0.91 | 0.81 |
| 20 | 2.75 | 3.14 | 3.9 |
| 21 | 3.22 | 4.72 | 5.72 |
| 22 | 3.74 | 11.74 | 14.86 |
| 23 | 2.7 | 4.82 | 6.98 |
| 24 | 3.51 | 3.56 | 4.32 |
| 25 | 4.07 | 0.83 | 1.02 |
| 26 | 1.72 | 0.83 | 1.2 |
| 27 | 1.44 | 0.97 | 1.73 |
| 28 | 1.99 | 1.29 | 1.83 |
| 29 | 3.75 | 1.98 | 2.24 |
| 30 | 1.32 | 3.3 | 3.62 |
| 31 | 1.44 | 7.76 | 9.43 |
| 32 | 1.6 | 13.86 | 18.03 |
| 33 | 1.1 | 6.35 | 9.17 |
| 34 | 1 | 3.81 | 2.41 |
| 35 | 0.88 | 3.51 | 1.2 |
| 36 | 0.8 | 1.95 | 0.65 |
