# Supplementary material for: Expression characteristics of pineal miRNAs at ovine different reproductive stages and the identification of miRNAs targeting the AANAT gene
Source: BMC Genomics. 2021 Mar 25;22:217. doi: 10.1186/s12864-021-07536-y (PMC7992348; doi:10.1186/s12864-021-07536-y)
Supplement: Supplementary file 2 — Additional file 2. Expression information of small RNA reads in ovine pineal gland. [file 12864_2021_7536_MOESM2_ESM.docx]

**Additional file 2. Expression information of small RNA reads in** **ovine pineal gland**

| Items | Stages | | |
| --- | --- | --- | --- |
|  | A | L | F |
| Raw reads | 12521961 | 19440216 | 11181419 |
| Reads after removing 3' adapter | 11186964 | 19099854 | 10629342 |
| Mapping rate of reads | 74.10% | 76.61% | 76.14% |
| Known miRNAs in miRbase | 63 | 87 | 84 |
| Predicated miRNAs | 131 | 271 | 265 |
| Total miRNAs | 194 | 358 | 349 |

A: anestrus; L: luteal phase; F: follicular phase.
