# Supplementary material for: Expression characteristics of pineal miRNAs at ovine different reproductive stages and the identification of miRNAs targeting the AANAT gene
Source: BMC Genomics. 2021 Mar 25;22:217. doi: 10.1186/s12864-021-07536-y (PMC7992348; doi:10.1186/s12864-021-07536-y)
Supplement: Supplementary file 3 — Additional file 3. The signaling pathways which the stage-specific expressed miRNAs were predominantly enriched in. [file 12864_2021_7536_MOESM3_ESM.docx]

**Additional file 3. The signaling pathways which the stage-specific expressed miRNAs were predominantly enriched in**

| **Signaling pathways in which anestrus-specific expressed miRNAs were predominantly enriched** | **Signaling pathways in which breeding season-specific expressed miRNAs were predominantly enriched** |
| --- | --- |
| MAPK signaling pathway - fly | MAPK signaling pathway - fly |
| Endocytosis | mTOR signaling pathway |
| mTOR signaling pathway | NOD-like receptor signaling pathway |
| MAPK signaling pathway | Apoptosis |
| Axon guidance | Axon guidance |
| Ras signaling pathway | MAPK signaling pathway |
| GnRH signaling pathway | Tight junction |
| Vascular smooth muscle contraction | Endocytosis |
| MAPK signaling pathway - yeast | Insulin signaling pathway |
| Regulation of actin cytoskeleton | AMPK signaling pathway |
| cAMP signaling pathway | Hippo signaling pathway |
| Tight junction | Olfactory transduction |
| Calcium signaling pathway | Lysosome |
| Phosphatidylinositol signaling system | MAPK signaling pathway - yeast |
| Adrenergic signaling in cardiomyocytes | Shigellosis |
| NOD-like receptor signaling pathway | Glycerophospholipid metabolism |
| Synaptic vesicle cycle | Taste transduction |
| Cholinergic synapse | alpha-Linolenic acid metabolism |
| cGMP - PKG signaling pathway | Oxytocin signaling pathway |
| Melanogenesis | Type II diabetes mellitus |
| Long-term potentiation | Adrenergic signaling in cardiomyocytes |
| Inositol phosphate metabolism | Rap1 signaling pathway |
| PI3K-Akt signaling pathway | Phosphatidylinositol signaling system |
| Insulin signaling pathway | Ras signaling pathway |
| Dopaminergic synapse | Dopaminergic synapse |
| Oxytocin signaling pathway | Vascular smooth muscle contraction |
| Circadian entrainment | GnRH signaling pathway |
|  | Cholinergic synapse |
|  | HTLV-I infection |
|  | Inflammatory mediator regulation of TRP channels |
|  | Carbon metabolism |
|  | Proteoglycans in cancer |
|  | Ribosome |
|  | Melanogenesis |
|  | Basal cell carcinoma |
|  | Morphine addiction |
|  | Estrogen signaling pathway |
|  | VEGF signaling pathway |
|  | Fatty acid degradation |
|  | Viral carcinogenesis |
|  | Fatty acid metabolism |
|  | Serotonergic synapse |
|  | Circadian entrainment |
|  | Glycerolipid metabolism |
|  | PPAR signaling pathway |
|  | Bacterial invasion of epithelial cells |
|  | Neurotrophin signaling pathway |
|  | Retrograde endocannabinoid signaling |
|  | Chemokine signaling pathway |
|  | Fc epsilon RI signaling pathway |
|  | Alcoholism |
|  | Vibrio cholerae infection |
|  | Calcium signaling pathway |
|  | GABAergic synapse |
|  | Adipocytokine signaling pathway |
|  | Cell adhesion molecules (CAMs) |
|  | Phototransduction - fly |
|  | Glutathione metabolism |
|  | Insulin secretion |
|  | beta-Alanine metabolism |
|  | ErbB signaling pathway |
|  | Thiamine metabolism |
|  | Fructose and mannose metabolism |
|  | Butanoate metabolism |
|  | Wnt signaling pathway |
