# Supplementary material for: Expression characteristics of pineal miRNAs at ovine different reproductive stages and the identification of miRNAs targeting the AANAT gene
Source: BMC Genomics. 2021 Mar 25;22:217. doi: 10.1186/s12864-021-07536-y (PMC7992348; doi:10.1186/s12864-021-07536-y)
Supplement: Supplementary file 4 — Additional file 4 The genes number and P value of pathways which target genes for differentially expressed miRNAs between anestrus and breeding season were enriched in. [file 12864_2021_7536_MOESM4_ESM.docx]

**Additional file 4. The genes number and *P* value of pathways which target genes for differentially expressed miRNAs between anestrus and breeding season enriched in.**

| **Pathways** | **Genes**  **number** | ***DE* Genes number** | ***P* value** |
| --- | --- | --- | --- |
| Endocytosis | 139 | 64 | 2.14E-11 |
| Axon guidance | 81 | 43 | 1.50E-10 |
| MAPK signaling pathway | 180 | 72 | 3.16E-09 |
| Vascular smooth muscle contraction | 84 | 38 | 4.34E-07 |
| Dopaminergic synapse | 74 | 34 | 1.12E-06 |
| Oxytocin signaling pathway | 104 | 39 | 6.93E-05 |
| GnRH signaling pathway | 58 | 24 | 0.00031 |
| cAMP signaling pathway | 137 | 46 | 0.00034 |
| Synaptic vesicle cycle | 39 | 18 | 0.00035 |
| Phosphatidylinositol signaling system | 36 | 17 | 0.00036 |
| Rap1 signaling pathway | 142 | 47 | 0.00043 |
| Ribosome | 114 | 39 | 0.00062 |
| Hippo signaling pathway | 103 | 36 | 0.00063 |
| PI3K-Akt signaling pathway | 219 | 66 | 0.00070 |
| Calcium signaling pathway | 130 | 43 | 0.00075 |
| Wnt signaling pathway | 90 | 32 | 0.00088 |
| Long-term potentiation | 40 | 17 | 0.00159 |
| cGMP - PKG signaling pathway | 108 | 36 | 0.00167 |
| Biosynthesis of amino acids | 44 | 18 | 0.00198 |
| GABAergic synapse | 58 | 22 | 0.00212 |
| Phototransduction - fly | 11 | 7 | 0.00254 |
| Phototransduction | 17 | 9 | 0.00348 |
| HIF-1 signaling pathway | 68 | 24 | 0.00413 |
| Protein digestion and absorption | 48 | 18 | 0.00598 |
| Estrogen signaling pathway | 64 | 22 | 0.00835 |
| Ubiquitin mediated proteolysis | 111 | 34 | 0.00970 |
