# Supplementary material for: Expression characteristics of pineal miRNAs at ovine different reproductive stages and the identification of miRNAs targeting the AANAT gene
Source: BMC Genomics. 2021 Mar 25;22:217. doi: 10.1186/s12864-021-07536-y (PMC7992348; doi:10.1186/s12864-021-07536-y)
Supplement: Supplementary file 5 — Additional file 5. The expression level of target genes for DE miRNAs between anestrus and breeding season in different reproductive stages. [file 12864_2021_7536_MOESM5_ESM.docx]

**Additional file 5. The expression level of target genes for DE miRNAs between anestrus and breeding season in different reproductive stages**

| **Pathway** | **Gene names** | **Anestrus** | **Luteal phase** | **Follicular phase** |
| --- | --- | --- | --- | --- |
| Axon guidance | SLIT3 | 9.93 | 12.61333333 | 21.87333333 |
| Axon guidance | MAPK1 | 7.943333 | 16.53333333 | 10.21666667 |
| Axon guidance | PLXNB2 | 8.726667 | 13.95 | 11.82 |
| Axon guidance | PAK2 | 5.133333 | 10.22666667 | 5.193333333 |
| Axon guidance | PIK3R3 | 6.136667 | 13.42 | 8.386666667 |
| Axon guidance | PAK4 | 7.073333 | 7.206666667 | 7.563333333 |
| Axon guidance | CAMK2B | 7.92 | 11.54 | 10.04666667 |
| Axon guidance | CDK5 | 39.86667 | 37.72333333 | 39.35 |
| Axon guidance | SEMA3B | 10.07667 | 13.57666667 | 11.17 |
| Axon guidance | GNAI2 | 30.34 | 53.72666667 | 35.22666667 |
| Axon guidance | DPYSL2 | 40.61667 | 67.26666667 | 48.19 |
| Axon guidance | RGS3 | 7.386667 | 11.08 | 8.593333333 |
| cAMP signaling pathway | RAPGEF3 | 7.713333 | 11.75666667 | 10.03666667 |
| cAMP signaling pathway | FXYD1 | 40.37 | 22.03333333 | 16.44666667 |
| cAMP signaling pathway | ATP2B4 | 7.266667 | 10.88666667 | 7.953333333 |
| cAMP signaling pathway | GABBR2 | 7.143333 | 14.33 | 9.373333333 |
| cAMP signaling pathway | NFKBIA | 13.39 | 22.32333333 | 13.06333333 |
| cAMP signaling pathway | PDE4A | 9.336667 | 15.27333333 | 13.44333333 |
| Circadian entrainment | PER1 | 4.61 | 10.45 | 6.693333333 |
| Circadian entrainment | RPS6KA5 | 10.47333 | 13.44666667 | 10.2 |
| Circadian entrainment | RYR1 | 18.28667 | 29.74 | 32.67 |
| Dopaminergic synapse | GNGT2 | 21.22 | 13.01333333 | 14.85 |
| Dopaminergic synapse | PPP2R5B | 16.4 | 19.70333333 | 17.21666667 |
| Dopaminergic synapse | PPP2R2C | 10.36667 | 25.97666667 | 15.63 |
| Dopaminergic synapse | PPP2R5D | 8.45 | 11.60666667 | 10.13333333 |
| Dopaminergic synapse | GNB2 | 30.13333 | 24.59666667 | 28.04 |
| Dopaminergic synapse | MAOB | 11.69667 | 14.44666667 | 8.096666667 |
| Dopaminergic synapse | GNB3 | 321.2 | 385.0966667 | 350.68 |
| Dopaminergic synapse | GNAO1 | 33.23667 | 49.52333333 | 28.30666667 |
| Dopaminergic synapse | GNA0 | 14.84333 | 36.93666667 | 17.84666667 |
| Dopaminergic synapse | GSK3A | 21.30333 | 29.80333333 | 24.69333333 |
| Dopaminergic synapse | DRD2 | 26.26 | 41.59666667 | 40.11333333 |
| Dopaminergic synapse | GNB1 | 210.2667 | 240.1666667 | 224.3233333 |
| Dopaminergic synapse | PPP2R5A | 9.326667 | 11.50666667 | 9.216666667 |
| Dopaminergic synapse | PPP2R2A | 9.013333 | 12.75333333 | 11.33333333 |
| Endocytosis | ARF2 | 8.273333 | 13.99666667 | 10.55333333 |
| Endocytosis | CHMP6 | 7.243333 | 7.763333333 | 8.783333333 |
| Endocytosis | PLD2 | 18.76667 | 21.74666667 | 18.64333333 |
| Endocytosis | VPS28 | 64.88333 | 38.09 | 54.69666667 |
| Endocytosis | ARFGEF1 | 21.94 | 21.44666667 | 20.67333333 |
| Endocytosis | HLA-B | 21.72333 | 14.96 | 18.20666667 |
| Endocytosis | ARPC1B | 18.21667 | 18.15666667 | 22.50333333 |
| Endocytosis | RAB11FIP3 | 8.32 | 12.09 | 12.03666667 |
| Endocytosis | SNX12 | 10.35 | 26.5 | 20.06 |
| Endocytosis | CDC42 | 6.793333 | 10.32 | 6.726666667 |
| Endocytosis | ARFGAP3 | 5.986667 | 10.21 | 7.003333333 |
| Endocytosis | DNM1 | 64.55333 | 86.34666667 | 76.08 |
| Endocytosis | SH3GLB2 | 25.54 | 34.55 | 31.83666667 |
| Endocytosis | RAB5B | 15.30333 | 29.64 | 18.42666667 |
| Endocytosis | ARPC5L | 13.19333 | 13.61333333 | 15.15 |
| Endocytosis | SH3GLB2 | 66.47667 | 57.52333333 | 69.93 |
| Endocytosis | SMAP2 | 8.866667 | 13.49666667 | 10.79333333 |
| Endocytosis | AP2M1 | 103.1933 | 143.32 | 118.48 |
| Endocytosis | PIP5K1A | 6.733333 | 13.15666667 | 7.463333333 |
| Endocytosis | CBLC | 5.66 | 7.246666667 | 6.733333333 |
| Endocytosis | CYTH2 | 24.30667 | 29.62333333 | 30.8 |
| Endocytosis | CHMP1A | 5.906667 | 8.346666667 | 7.39 |
| Endocytosis | AP2S1 | 107.87 | 70.54666667 | 93.88 |
| Endocytosis | CHMP2A | 205.8633 | 115.16 | 162.9433333 |
| Endocytosis | VPS4A | 22.96667 | 36.94 | 30.48333333 |
| Endocytosis | AP2A1 | 14.75667 | 16.98333333 | 15.75 |
| Endocytosis | ARAP1 | 8.26 | 11.37666667 | 10.98333333 |
| Endocytosis | ARRB1 | 7.103333 | 18.80666667 | 13.16333333 |
| Endocytosis | CHMP4B | 85.24333 | 99.97666667 | 90.70666667 |
| Endocytosis | ITCH | 45.64667 | 44.15333333 | 49.51666667 |
| Endocytosis | ARF5 | 25.30333 | 24.90333333 | 28.67 |
| Endocytosis | AGAP3 | 17.01 | 19.04666667 | 17.56 |
| Endocytosis | RAB7A | 50.51 | 65.48 | 50.47 |
| Endocytosis | ACAP3 | 6.346667 | 15.26333333 | 11.70666667 |
| Endocytosis | CAPZB | 40.5 | 47.22 | 43.91666667 |
| Endocytosis | ARPC2 | 20.05 | 22.99666667 | 25.5 |
| Endocytosis | KIF5C | 7.463333 | 13.22666667 | 10.57666667 |
| Endocytosis | ARF1 | 59.74 | 107.5866667 | 75.61666667 |
| Endocytosis | EPS15L1 | 10.28333 | 16.45 | 13.45333333 |
| Endocytosis | RAB11B | 51.5 | 87.07666667 | 59.22333333 |
| Endocytosis | DNM2 | 9.77 | 17.21 | 13.08 |
| GnRH signaling pathway | GRB2 | 29.29 | 39.92 | 40.43666667 |
| GnRH signaling pathway | PTK2B | 5.186667 | 8.22 | 7.4 |
| GnRH signaling pathway | MAP3K4 | 5.076667 | 8.376666667 | 6.703333333 |
| Phototransduction | RCVRN | 291.48 | 156.83 | 299.35 |
| Phototransduction | PDE6G | 52.03667 | 54.39666667 | 38.57 |
| Phototransduction | RGS9 | 11.61333 | 27.57333333 | 20.39333333 |
| Phototransduction | SLC24A1 | 39.58 | 41.95666667 | 48.99333333 |
| Phototransduction | GUCA1A | 621.48 | 453.7433333 | 433.5633333 |
| Protein digestion and absorption | SLC3A2 | 67.03333 | 61.22666667 | 71.08333333 |
| Protein digestion and absorption | SLC8A3 | 7.22 | 13.00666667 | 8.986666667 |
| Protein digestion and absorption | COL4A1 | 6.18 | 7.593333333 | 7.33 |
| Protein digestion and absorption | COL5A3 | 23.18333 | 43.54 | 46.82666667 |
| Vascular smooth muscle contraction | RAMP2 | 114.3133 | 86.09333333 | 101.31 |
| Vascular smooth muscle contraction | CACNA1F | 8.213333 | 12.21 | 12.11 |
| Vascular smooth muscle contraction | MYL6 | 275.0833 | 271.08 | 308.54 |
| Vascular smooth muscle contraction | ACTG2 | 2.943333 | 3.643333333 | 2.913333333 |
| Vascular smooth muscle contraction | CALM1 | 149.1 | 173.0766667 | 147.7966667 |
| Vascular smooth muscle contraction | RAMP1 | 59.30667 | 26.41 | 14.79666667 |
| Vascular smooth muscle contraction | CALM1 | 186.26 | 179.7666667 | 186.8466667 |
| Vascular smooth muscle contraction | ARHGEF1 | 7.18 | 8.426666667 | 7.816666667 |
| Vascular smooth muscle contraction | Uncharacterized | 9.933333 | 13.52666667 | 13.30666667 |
| Vascular smooth muscle contraction | NPR2 | 7.54 | 12.75333333 | 10.56 |
| Vascular smooth muscle contraction | ADRA1B | 14.06333 | 19.34333333 | 14.78666667 |
| Vascular smooth muscle contraction | MAP2K2 | 43.06667 | 36.5 | 40.45 |
| Ribosome | RPL30 | 8.593333 | 7.336666667 | 5.14 |
| Ribosome | RPL4 | 0.653333 | 0.546666667 | 0.296666667 |
| Ribosome | RPLP2 | 408.9 | 239.8233333 | 138.27 |
| Ribosome | RPLP1 | 1935.22 | 1415.99 | 1102.466667 |
| Ribosome | MRPL2 | 13.5 | 10.38666667 | 11.75 |
| Ribosome | RPL10A | 0.333333 | 0.216666667 | 0.143333333 |
| Ribosome | RPL35 | 395.9167 | 165.1333333 | 86.30333333 |
| Ribosome | RPL17 | 0.83 | 0.076666667 | 0.143333333 |
| Ribosome | RPS21 | 93.25667 | 0 | 0 |
| Ribosome | MRPS15 | 34.35667 | 24.58333333 | 32.58666667 |
| Ribosome | RPL13A | 237.2867 | 173.44 | 188.5566667 |
| Ribosome | RPL28 | 114.17 | 87.51666667 | 74.66666667 |
| Ribosome | RPS5 | 265.1933 | 145.35 | 173.8366667 |
| Ribosome | RPS11 | 1631.583 | 1597.073333 | 1533.656667 |
| Ribosome | RPS13 | 218.3167 | 106.82 | 121.1366667 |
| Ribosome | RPS25 | 1.746667 | 0.943333333 | 0.88 |
| Ribosome | RPSA | 305.76 | 155.8766667 | 169.8333333 |
| Ribosome | RPL34 | 5.13 | 2.726666667 | 0.406666667 |
| Ribosome | MRPL18 | 69.81 | 42.44666667 | 61.71666667 |
| Ribosome | RPL10 | 0.033333 | 0 | 0 |
| Ribosome | RPL5 | 33.02333 | 29.04666667 | 28.32666667 |
| Ribosome | RPL18A | 265.13 | 118.16 | 163.4633333 |
