# Supplementary material for: Expression characteristics of pineal miRNAs at ovine different reproductive stages and the identification of miRNAs targeting the AANAT gene
Source: BMC Genomics. 2021 Mar 25;22:217. doi: 10.1186/s12864-021-07536-y (PMC7992348; doi:10.1186/s12864-021-07536-y)
Supplement: Supplementary file 7 — Additional file 7. Highly expressed genes during anestrus or breeding season in pineal gland of sheep. [file 12864_2021_7536_MOESM7_ESM.docx]

**Additional file 7. Highly expressed genes during anestrus or breeding season in pineal gland of sheep**

| **Gene_names** | **Loci** | **Expression level of each stage** | | | | |  | |  |
| --- | --- | --- | --- | --- | --- | --- | --- | --- | --- |
|  |  | **Anestrus** | **Luteal phase** | **Follicular**  **phase** | | **Functions** | | |  |
| **Highly expressed genes in anestrus** | | | | | | | |  |  |
| RPS15A | chr13:60128612-60129005 | 67.8666 | 5.37522 | | 2.58734 | K02957 small subunit ribosomal protein S15Ae | | | |
| RPS9 | chr24:30822890-30823442 | 32.3854 | 2.02744 | | 2.61030 | K02997 small subunit ribosomal protein S9e | | | |
| TIMP1 | chrX:53791005-53843432 | 295.113 | 28.0347 | | 36.1622 | SHEEP Metalloproteinase inhibitor 1 | | | |
| Uncharacterized | chr1:98629592-98706395 | 15.4215 | 0.11180 | | 0.09425 | / | | | |
| HSD3B | chr1:96023032-96101562 | 32.6801 | 0.13737 | | 0.18120 | K00070 3beta-hydroxy-delta5-steroid dehydrogenase | | | |
| STAR | chr26:31960576-31966284 | 18.5287 | 0.41952 | | 0.71832 | SHEEP Steroidogenic acute regulatory protein | | | |
| SCAND3 | chr20:29180207-29263651 | 12.3573 | 1.28555 | | 0.91891 | K02998 small subunit ribosomal protein SAe | | | |
| LMF1 | chr24:626649-719495 | 220.512 | 28.3751 | | 18.4845 | K04547 guanine nucleotide binding protein gamma 13 | | | |
| **Highly expressed genes during breeding season** | | | | | | | | | |
| GABARAPL2 | chr14:2251814-2262220 | 1093.32 | 4935.25 | | 11478.4 | K08341 GABA(A) receptor-associated protein | | | |
| MT-CO1 | chrX:56345528-56345705 | 5980.01 | 64164.3 | | 68682.2 | K02256 cytochrome c oxidase subunit I | | | |
| CCDC23 | chr1:17059212-17064233 | 92.2275 | 1059.02 | | 1230.55 | BOVIN Coiled-coil domain-containing protein | | | |
| TBCA | chr7:8632923-8646584 | 620.719 | 3535.68 | | 8636.83 | BOVIN Tubulin-specific chaperone A | | | |
| CRHBP | chr7:7910103-7922061 | 1.23766 | 35.9904 | | 20.8085 | SHEEP Corticotropin-releasing factor-binding protein | | | |
| Uncharacterized | chr1:261893431-261897406 | 34.1699 | 380.497 | | 581.133 | / | | | |
| MRPL54 | chr5:17772941-17773875 | 304.536 | 2454.87 | | 6408.15 | BOVIN 39S ribosomal protein L54 | | | |
| Uncharacterized | chr3:203489227-203489422 | 27.8743 | 721.354 | | 623.281 | / | | | |
| ACYP2 | chr3:69651113-69818058 | 393.921 | 3106.78 | | 8907.16 | K01512 acylphosphatase | | | |
| Uncharacterized | chr23:22415930-22434803 | 12.4264 | 413.838 | | 329.615 | / | | | |
| NDUFC1 | chr17:18057147-18059636 | 230.505 | 1730.10 | | 6557.56 | K03967 NADH dehydrogenase (ubiquinone) 1 subcomplex unknown 1 | | | |
| CFDP2 | chr23:42004575-42004833 | 17.6585 | 212.270 | | 508.066 | TRAJA Craniofacial development protein 2 | | | |
| TMEM167B | chr1:85579244-85580305 | 17.2583 | 440.775 | | 513.009 | K09532 DnaJ homolog subfamily C member 12 | | | |
| PKIB | chr8:15590256-15595873 | 106.389 | 1127.7 | | 3362.53 | HUMAN cAMP-dependent protein kinase inhibitor beta | | | |
| Uncharacterized | chr15:38429434-38429632 | 86.6497 | 1901.53 | | 3663.08 | / | | | |
| Uncharacterized | chr15:25554148-25556279 | 34.5828 | 682.748 | | 1616.40 | / | | | |
| SUMO3 | chr1:262782250-262786461 | 304.301 | 7786.45 | | 22465.7 | K12160 small ubiquitin-related modifier | | | |
| EYA3 | chr2:238171229-238265280 | 1.82441 | 3.37734 | | 2.25648 | K01104 protein-tyrosine phosphatase | | | |
| EYA1 | chr9:47588480-47742886 | 0.15434 | 0.65968 | | 0.19639 | K01104 protein-tyrosine phosphatase | | | |
| EYA2 | chr13:75146640-75328455 | 0.83905 | 2.15050 | | 1.13234 | K01104 protein-tyrosine phosphatase | | | |
| EYA4 | chr8:58476171-58891338 | 0.23395 | 0.50439 | | 0.36680 | K01104 protein-tyrosine phosphatase | | | |
