# Supplementary material for: Expression characteristics of pineal miRNAs at ovine different reproductive stages and the identification of miRNAs targeting the AANAT gene
Source: BMC Genomics. 2021 Mar 25;22:217. doi: 10.1186/s12864-021-07536-y (PMC7992348; doi:10.1186/s12864-021-07536-y)
Supplement: Supplementary file 8 — Additional file 8. Predicted miRNAs potentially targeting genes in ribosome pathway. [file 12864_2021_7536_MOESM8_ESM.docx]

**Additional file 8. Predicted miRNAs potentially targeting genes in ribosome pathway**

| **Genes** | **miRNA ID** | **Homolog ID** |
| --- | --- | --- |
| RPLP2 | miR-19 | osa-miR396e-5p |
| RPLP2 | miR-108 | rno-miR-328a-3p |
| RPLP1 | miR-109 | mmu-miR-326-3p |
| RPL35 | miR-108 | rno-miR-328a-3p |
| RPL35 | miR-109 | mmu-miR-326-3p |
| RPL35 | miR-66 | mmu-miR-129-1-3p |
| RPL35 | oar-miR-370-5p | - |
| RPS5 | miR-395 | oan-miR-1388-3p |
| RPS5 | miR-59 | ssc-miR-296-3p |
| RPS5 | oar-miR-150 | - |
| RPS5 | oar-miR-370-5p | - |
| RPS13 | miR-32 | rno-miR-324-5p |
| RPSA | miR-59 | ssc-miR-296-3p |
| RPSA | oar-let-7i | - |
| RPL18A | miR-5 | hsa-miR-27a-3p |
| RPL18 A | oar-miR-27a | - |
