# Supplementary material for: Expression characteristics of pineal miRNAs at ovine different reproductive stages and the identification of miRNAs targeting the AANAT gene
Source: BMC Genomics. 2021 Mar 25;22:217. doi: 10.1186/s12864-021-07536-y (PMC7992348; doi:10.1186/s12864-021-07536-y)
Supplement: Supplementary file 9 — Additional file 9 Stem-loop primers employed for RT-PCR of miRNAs and primers employed for Q-PCR of AANAT and miRNAs. [file 12864_2021_7536_MOESM9_ESM.docx]

**Additional file 9. Stem-loop primers employed for RT-PCR of miRNAs and primers employed for Q-PCR of *AANAT* and miRNAs**

| **Name** | **Primer sequences (5’-3’)** |
| --- | --- |
| miR-89 RT | GTCGTATCCAGTGCAGGGTCCGAGGTATTCGCACTGGATACGACCTGCCTGT |
| miR-89 F | ACAGCAGGCACAGACAGC |
| miR-89 R | GCAGGGTCCGAGGTATTC |
| miR-201 RT | GTCGTATCCAGTGCAGGGTCCGAGGTATTCGCACTGGATACGACAGGCGGAC |
| miR-201 F | CGGCTTATTCCCACTCC |
| miR-201 R | GCAGGGTCCGAGGTATT |
| miR-919 RT | GTCGTATCCAGTGCAGGGTCCGAGGTATTCGCACTGGATACGACTCCCACGA |
| miR-919 F | GAGGGTTTGGGTTTGGTC |
| miR-919 R | GCAGGGTCCGAGGTATTC |
| miR-922 RT | GTCGTATCCAGTGCAGGGTCCGAGGTATTCGCACTGGATACGACTGGCCCGG |
| miR-922 F | ATTATTCCCCCCACGC |
| miR-922 R | GCAGGGTCCGAGGTATT |
| miR-925 RT | GTCGTATCCAGTGCAGGGTCCGAGGTATTCGCACTGGATACGACACCCTCCC |
| miR-925 F | CGGTATTGGGCAGGGTT |
| miR-925 R | GCAGGGTCCGAGGTATT |
| U6 F | CTCGCTTCGGCAGCACA |
| U6 R | AACGCTTCACGAATTTGCGT |
| AANAT-F | GCTGGTGCCCTTTTACCAGA |
| AANAT-R | CTGGGGATTGAGTAGGAGCC |
| GAPDH-F | GAGAAACCTGCCAAGTATGA |
| GAPDH-R | CGAAGGTAGAAGAGTGAGTG |

Primers with “RT” in the name: Stem-loop primers employed for RT-PCR of miRNA; primers with “F” or “R” in the name: forward or reverse primers employed for Q-PCR of miRNA and genes.
