# Supplementary material for: Expression characteristics of pineal miRNAs at ovine different reproductive stages and the identification of miRNAs targeting the AANAT gene
Source: BMC Genomics. 2021 Mar 25;22:217. doi: 10.1186/s12864-021-07536-y (PMC7992348; doi:10.1186/s12864-021-07536-y)
Supplement: Supplementary file 10 — Additional file 10. Mature sequences of miRNAs and inserted sequences in the pcDNA6.2-GW/EmGFP-miR vector. [file 12864_2021_7536_MOESM10_ESM.docx]

**Additional file 10. Mature sequences of miRNAs and inserted sequences in the pcDNA6.2-GW/EmGFP-miR vector**

| **Sequence name** | **Sequence (5’-3’)** |
| --- | --- |
| **Mature Sequence of miR-89** | ACAGCAGGCACAGACAGGCAG |
| Inserted sequences (miR-89-F) | TGCTGACAGCAGGCACAGACAGGCAGGTTTTGGCCACTGACTGACCTGCCTGTGTGCCTGCTGT |
| Inserted sequences (miR-89-R) | CCTGACAGCAGGCACACAGGCAGGTCAGTCAGTGGCCAAAACCTGCCTGTCTGTGCCTGCTGTC |
| **Mature Sequence of miR-201** | UUCCCACUCCCUCUGUCCGCCU |
| Inserted sequences (miR-201-F) | TGCTGTTCCCACTCCCTCTGTCCGCCTGTTTTGGCCACTGACTGACAGGCGGACAGGGAGTGGGAA |
| Inserted sequences (miR-201-F) | CCTGTTCCCACTCCCTGTCCGCCTGTCAGTCAGTGGCCAAAACAGGCGGACAGAGGGAGTGGGAAC |
| Negative control-F | TGCTGAAATGTACTGCGCGTGGAGACGTTTTGGCCACTGACTGACGTCTCCACGCAGTACATTT |
| Negative control-R | CCTGAAATGTACTGCGTGGAGACGTCAGTCAGTGGCCAAAACGTCTCCACGCGCAGTACATTT |
